# Suicide Considered a Mental Epidemic

**Published:** 1897-01

**Authors:** Forbes Winslow

**Affiliations:** London, Vice-President of Medico-Legal Congress, Chairman Department of Mental Medicine


### Abstracts and Selections.

### Suicide Considered a Mental Epidemic.

DR. FORBES WINSLOW, OF LONDON, VICE-PRESIDENT OF MEDICO-LEGAL CONGRESS, CHAIRMAN DEPARTMENT OF MENTAL MEDICINE.

With reference to suicide, there is no fact that has been more clearly established than that of its hereditary character. Of all diseases to which the various organs are subject, there are none more naturally transmitted from one generation to another than affections of the brain. It is not necessary that the disposition to suicide should manifest itself in every generation. It often passes over one, and appears in the next, like insanity unattended with this propensity. But if the members of the family so predisposed are carefully examined, it will be found that the various shades and gradations of the malady will be easily perceptible. Some are distinguished for their flightiness of manner, others for their strange eccentricity, likings, and dislikings, irregularity of their passions, capricious, and excitable temperament, hyprochondriasis, and melancholia. These are often but the minute shades and variations of a hereditary disposition to suicidal madness.

A gentleman suddenly, and without any apparent reason, cut his throat. The father had always been a man of strong passions, easily aroused, and when so, was extremely violent. The brother was a man of impulse; he always acted by fits and starts, and therefore never could be depended upon. The sister had a strange, unnatural, and superstitious horror of particular colors and odors. A yellow dress caused a feeling approaching to syncope, and the smell of hay produced great nervous excitement. The grandfather had been convicted of homicide, and had been confined for two years in a madhouse. Andral relates the case of a father who died from the effects of disease of the brain; the mother died sane. They had six children, three boys and three girls. Of the hoys, the oldest was a man of original mind; the second was very extravagant in his habits, and was ultimately confined in a madhouse; the third was extremely violent in his temper. Of the girls, one had fits of apoplexy; one became insane; the third died of cholera, not, however, until she exhibited indications of mental aberration.

A case more singular than the last is recorded. All the members of a particular family being hereditarily disposed, exhibited, when they arrived at a certain age, a desire to commit self-destruction. It required no exciting cause to develop the fatal disposition. No wish was expressed, nor attempt made to overpower the suicidal inclination, and the greatest industry and ingenuity were exercised by the parties to effect their purpose. In two cases the propensity was subdued by proper medical and moral treatment, but, just in proportion to its being suppressed did the idea of suicide appear to fix itself resolutely in the mind. The desire came upon the individual like the attacks of intermittent fever.

A. K., a man aged fifty-seven, was twice married. He was a shoemaker by trade, but, not having received any education, his wife was compelled to keep all his accounts. He had experienced, when young, a blow on the head, which occasionally gave him pain. He became very intemperate in his habits, and at particular intervals he exhibited an uncontrollable temper, quarreled with everybody, neglerted his business, abused his wife, and became extravagant and melancholy. During the paroxysm he would exclaim: "Oh. my unlucky head. I am again a lost man!" When the attack subsided he returned to his business, was affectionate to his wife and family, most humbly begged for her pardon for having ill-treated her, and expressed the greatest contrition for his conduct. These attacks came at regular intervals. He procured a piece of rope for the purpose of hanging himself, and for some months carried it about with him in his pocket for that purpose. During one of his fits he effected his object. His grandfather had strangled himself, and his brother and sister had attempted suicide.

A common cause of suicide is a feeling of false pride, Owing to the ridiculously false views which are taken of worldly honors, the idea which a sickly sentimentality infuses into the mind, this feeling is engendered, to an alarming extent, through the different ranks of society. This constitutes one great element which is undermining and disorganizing our social condition. A fictitious value is affixed to wealth and position in the world; it is estimated for itself alone, all other considerations being placed out of view.

We cannot conceive how this evil is to be obviated, unless it be possible to revolutionize the ideas which are generally attached to fame and worldly grandeur. It is difficult to persuade such persons that the end of fame is merely

> "To have, when the original is dust,
> A name, a wretched picture, and worse—bust."

There is a nameless, undefinable something, that the world is taught to sigh after, is always in search of, a moral *ignis fatuus*, which is dazzling to lead it from the road which points to true and unsophisticated happiness.

Among the causes which operate in producing the disposition to commit suicide, we must not omit to mention those connected with erroneous religious notions. M. Falret justly remarks that the religious systems of the Druids, Odin, and Mahommed, by inspiring a contempt for death, have made many suicides. The man who believes that death is an eternal sleep scorns to hold up against calamity, and prefers annihilation. The skeptic also often frees himself by self-destruction from the agony of doubting. The maxim of the Stoics, that the man should live only so long as he ought, not so long as he is able, is, we may observe, the very parent of suicide.

The Brahmin, looking on death as the very entrance into life, and thinking a natural death dishonorable, is eager, at all times, to get rid of life. The Epicureans and Paripatetics ridiculed suicide as being death caused by fear of death.

M. Falret, however, goes perhaps too far when he asserts that the noble manner in which the gladiators died in public not only familiarized the Romans with death, but rendered the thoughts of it rather agreeable than otherwise.

Misinterpretations of passages of Scripture will sometimes lead those who are piously inclined to commit suicide. M. Gillot hung himself at the age of seventy-five, having left, in his own handwriting, the following apology: "Jesus Christ has said that when a tree is old, and can no longer bear fruit, it is good that it should be destroyed." (He had more than once attempted his life before the fatal act.) Dr. Burrows attended a nobleman who, for fear of being poisoned, though he pretended it was in imitation of our Savior's fast, took nothing but strawberries and water for three weeks, and these in very moderate quantities. He never voluntarily abandoned his resolution. He was at length compelled to take some nutriment, but not until inanition had gone too far; and he died completely attenuated. When sound religious principles produce a struggle in the mind which is beginning to aberrate, the contest generally ends in suicide.

Among the causes of suicide, the foggy climate of England has been brought prominently forward. The spacious and inaccurate conclusions of Montesquieu on this point have mislead the public mind. The climate of Holland is much more gloomy than that of England, and yet in that country suicide is by no means common. From the following tabular statement we see that the popular notion of the month of November, being the "suicide month," is founded on erroneous data. The average number of suicides in each month, for years, may taken as follows:

| | | | |
|---|---|---|---|
| January | 213 | July | 301 |
| February | 218 | August | 296 |
| March | 275 | September | 246 |
| April | 374 | October | 198 |
| May | 328 | November | 131 |
| June | 336 | December | 217 |
| Total | | | 3,133 |

It has been clearly established that in all the European capitals, when anything approaching to correct statistical evidence can be procured, the maximum of suicide is in the months of June and July, the minimum in October and November. Temperature appears to exercise a much more decided influence than the circumstances of moisture and dryness, storms or serenity.

M. Villeneuve has observed a warm, humid, and cloudy atmosphere to produce a marked bad effect at Paris, and that so long as the barometer indicated stormy weather this effect continued. Contrary, however, to the opinion of Villeneuve, it appears that by far the fewer number of suicides occur in the autumn and winter at Paris than in the spring and summer.

When the thermometer of Fahrenheit ranges from 80° to 90°, suicide is most prevalent. The English have been accused of being the beau-ideal of suicide people. The charge is almost too ridiculous to merit serious refutation. Suicide is not an offense that can be deemed cognizable by the civil magistrate. It is to be considered a sinful and vicious action. To punish suicide as a crime is to commit a solecism in legislation. The unfortunate individual, by the very act of suicide, places himself beyond the vengeance of the law. He has anticipated its operation. He has rendered himself amenable to the highest tribunal, namely, that of his creator. No penal enactments, however stringent, can affect him. What is the operation of the law under these circumstances? A verdict of felo de se is returned, and the innocent relatives of the suicide are disgraced and branded with infamy, and that, too, on evidence of an ex parte nature. It is unjust, inhuman, unnatural, and unchristian that the law should punish the innocent family of the man who, in a moment of frenzy, terminates his own miserable existence. It was clearly established before the alteration of the law respecting suicide, the fear of being buried in a cross-road, and having a stake driven through the body, had no beneficial effect in decreasing the number of suicides; and the verdict of felo de se now occasionally returned, is productive of no advantage whatever, and only injures the surviving relatives.

If the view which we have taken of the cause of suicide be a correct one, no stronger argument can be urged for the impropriety of bringing the strong arm of the law to bear upon those who court a voluntary death. In the majority of cases it will be found that some heavy calamity has fastened itself upon the mind, and the spirits have been extremely depressed. The individual loses all pleasure in society, hope vanishes, and despair renders life intolerable, and death an apparent relief. The evidence, which is generally submitted to a coroner's jury, is of necessity imperfect; and although the suicide may, to all appearance be in possession of his right reason, and have exhibited, at the moment of killing himself, the greatest calmness, coolness, and self-possession, this would not justify the coroner or the jury in concluding that derangement of mind was not present.

If the mind be overpowered by "grief, sickness, infirmity, or other accident," as Sir Matthew Hale expresses it, the law presumes the existence of lunacy. Any passion that powerfully exercises the mind, and prevents the reasoning faculty from performing its duty, causes temporary derangement. It is not necessary, in order to establish the presence of insanity, to prove the person to be laboring under a delusion of intellect—a false creation of mind. A man may allow his imagination to dwell upon an idea until it acquires an unhealthy ascendency over intellect, and in this way a person may commit suicide from an habitual belief in the justifiableness of the act. If a man, by a distorted process of reasoning, argues himself into a conviction of the propriety of adopting a particular course of conduct without any reference to the necessary result of that train of thought, it is certainly no evidence of his being in possession of a sound mind. A person may reason himself into a belief that murder, under certain circumstances not authorized by the law, is perfectly just and proper. The circumstances of his allowing his mind to reason on the subject, is a prima facie case against his sanity. At least it demonstrates a great weakness of the moral constitution. A man's morals must be in an imperfect state of development who reasons himself into the conviction that self-murder is under any circumstances jutifiable.

We dwell at some length on this subject, because we feel assured that juries do not pay sufficient attention to the influence of passion in overclouding the understanding. If the notion, that in every case of suicide the intellectual or moral faculties are perverted, be generally received, it will at once do away with the verdict of felo de se. Should the jury entertain a doubt as to the presence of derangement—and such cases may present themselves—it is their duty, in accordance with the well-known principle of British jurisprudence, to give the person the benefit of that doubt, and thus a verdict of lunacy may be conscientiously returned in every case of this description.

Having, we think, clearly established that no penal law can act beneficially in preventing self-destruction—first, because it would punish the innocent for the crimes of the guilty, and, secondly, that, owing to insanity being present in every instance, the person determined on suicide is indifferent as to the consequences of his action—it becomes our province to consider what are the legitimate means of staying the progress of an offense that undermines the foundation of society and social happiness.

If we are justified in maintaining that the majority of the cases of suicide result from a vitiated condition of the moral principle, then it is certainly a legitimate mode of preventing the commission of the offense to elevate the character of man as a moral being. It is no legitimate argument against this position to maintain that insanity, in all its phases, marches side by side with civilization and refinement; but it must not be forgotten that a people may be refined and civilized, using these terms in their ordinary signification, who have not a just conception of their duties as members of a christian community. Let the education of the heart go side by side with the education of the head; inculcate the ennobling thought, that we live not for ourselves, but for others; that it is an evidence of true christian courage to face bravely the ills life, to bear with impunity "the whips and scorns of time, the oppressor's wrong, and the proud man's contumely," and we disseminate principles which will give expansion to those faculties that alone can fortify the mind against the commission of a crime alike repugnant to all human and divine laws,

"And make us rather bear the ills we have,
Than fly to others that we know not of."

—*Medico-Legal Journal*, *Sept.*, *1896.*

